# Immobilization of PETase enzymes on magnetic iron oxide nanoparticles for the decomposition of microplastic PET†

**DOI:** 10.1039/d1na00243k

**Published:** 2021-06-14

**Authors:** Sebastian P. Schwaminger, Stefan Fehn, Tobias Steegmüller, Stefan Rauwolf, Hannes Löwe, Katharina Pflüger-Grau, Sonja Berensmeier

**Affiliations:** Department of Mechanical Engineering, Bioseparation Engineering Group, Technical University of Munich Garching Germany s.schwaminger@tum.de s.berensmeier@tum.de; Department of Mechanical Engineering, Systems Biotechnology, Technical University of Munich Garching Germany

## Abstract

Polyethylene terephthalate (PET) is responsible for a large amount of environmental contamination with microplastics. Based on its high affinity, the PET degrading enzyme PETase can be immobilized on superparamagnetic iron oxide nanoparticles through a His-tag. The His-tag increases enzyme stability, and allows magnetic separation for recovery. Multiple recycling steps are possible and microplastic particles can be decomposed depending on the PET's crystallinity. The separation or decomposition of PET allows for a sustainable way to remove microplastic from water.

## Abstract 2

The enzyme PETase can be attached to superparamagnetic iron oxide nanoparticles through a His-tag facilitating a high enzymatic activity for the degradation of PET microplastic particles.

## Graphical abstract

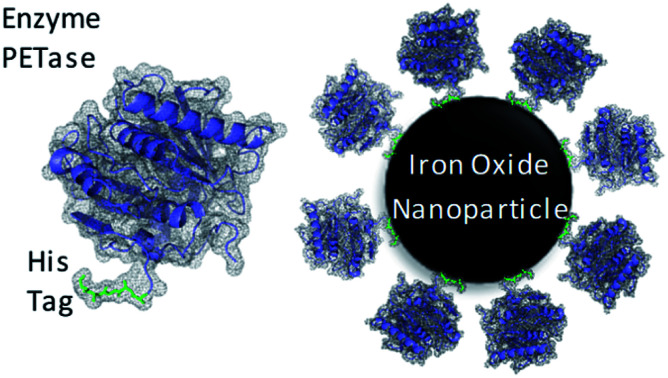

## Introduction

Steadily increasing amounts of microplastic particles in the environment represent a burden for biological organisms worldwide.^1^ The size of these particles ranges between nanometers and micrometers up to 5 mm. These microparticles made of plastics are referred to as microplastics (MP).^2,3^ Due to their chemical stability, MPs can last for a long time in the environment. In the case of polyethylene terephthalate (PET), it can be up to 450 years.^4^ Aside from tire wear, which causes 28% of the MPs pollution, the clothing industry accounts for the highest microplastic emissions.^5^ During washing processes, many fibers from polyester clothes are released into the oceans through wastewater. These fibers make up about 35% of the microplastic pollution worldwide.^5,6^ Apart from polyester clothes, PET is mainly used as a raw material in drinking bottle manufacturing.^7^ Furthermore, oven-ready metal trays, cable lining, and other household products, such as toothpastes, scrubs, shampoos, and body cleanser can be manufactured from PET and lead to the emission of microplastics.^8^ In the case of aerial uptake, MPs can act as carriers for pathogens or other chemical substances which suggest a potential health risk.^9–11^ In general, particles smaller than 10 μm are considered more problematic as they can diffuse directly into the lungs and cause inflammation.^12^ However, there is little research and therefore missing knowledge on health consequences regarding MP.^12–14^

Strong acids and bases can hydrolyze PET, which also affects the crystallinity of the material.^15^ Chemical recycling of PET is possible by solvolysis and pyrolysis.^16–18^ However, these methods of recycling lead to the generation of pollutants, and therefore, more environmentally friendly methods are needed.^19,20^ Several microorganisms possess the ability to decompose PET into its monomers terephthalic acid (TPA) and ethylene glycol (EG).^7,21,22^ The PET degrading cutinase from *Ideonella sakaiensis*, is so far known as the most efficient cutinase operating at room temperature, which decomposes PET to EG and TPA.^4,21,23,24^ Several factors such as temperature, pH and ionic strength influence enzymatic activity of PETase.^21,25^ Enzymes derived from *Ideonella sakaiensis* have been tested towards homogeneous substrate materials such as PET bottle walls or films.^26,27^ The crystallinity of PET is an important parameter directly influencing enzymatic activity.^21,26^

The immobilization of enzymes on solid carriers is a possibility to overcome the challenges of protein production and recycling.^28^ One objective of immobilization is increasing the enzyme's stability against environmental impacts such as temperature or pH changes while keeping the highest possible catalytic activity of the enzyme and eventually allowing for a more effective process handling and enzyme reuse.^29,30^ There are different kinds of immobilization techniques depending on the interaction mechanisms between carrier and enzymes. Conventional interactions include covalent and non-covalent adsorption and deposition, ionic interaction, cross-linking, encapsulation in gels and bio-conjugation.^29,31^ While all methods have their advantages and disadvantages, the directed immobilization *via* a selective peptide tag seems most appealing.^32^ As an enzyme carrier, magnetic nanoparticles (MNPs) stand out by their chemical, physical and especially superparamagnetic properties.^31,33,34^ The magnetic properties allow to separate MNPs from their surrounding medium by inducing a magnetic field. Another advantage of nanoscale carriers is the large specific surface area and low steric hindrance compared to porous materials.^35^ Furthermore, the manufacturing of iron oxide nanoparticles is comparatively cost-efficient^35^ and they are generally recognized as safe (GRAS).^36^ Hence, they are a suitable solid carrier material for enzymatic immobilization purposes.^32,37,38^ Our objective was to create a novel nano-biocatalyst (NBC) with iron oxide nanoparticles as solid material carriers and the PET degrading PETase on its surface. To this end, we employed the affinity of the known histidine tag for MNPs to immobilize the enzyme PETaseS238F/W159H.^39^ Furthermore, we analyzed in detail the crystallinity change induced by enzymatic degradation of a powder-like PET substrate. The here presented NBC system is supposed to combine immobilization advantages, such as increasing durability and stability, with a still remanent high enzymatic activity compared to the pure enzyme. In addition, the magnetic separation feature of magnetic nanoparticles allows for a high separability and recyclability grade for a straightforward process handling.

## Results and discussion

**Fig. 1:**
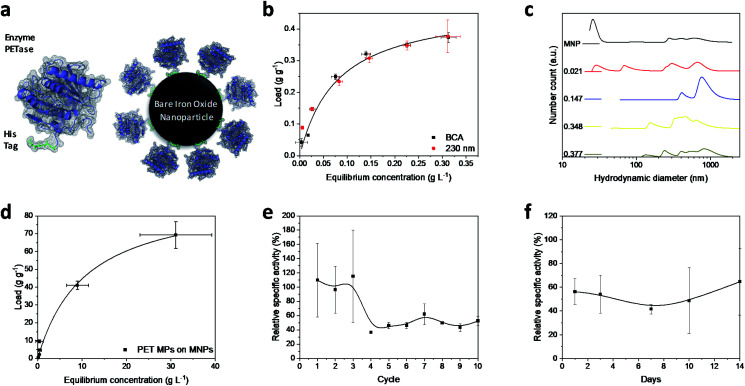
(a) Schematic illustration of PETase attachment through a His tag to MNPs. (b) Adsorption isotherms at different PETase concentrations detected photometrically at 230 nm (red dots) or by means of a BCA-assay (black squares). Both experiments were conducted in 50 mM Tris buffer at pH 7.5. The error bars were derived from three incubation experiments and supernatant analysis in triplicates (±SD). (c) Hydrodynamic diameter of NBCs with different loads. The enzyme loads vary from 0 to 0.377 g g^−1^ at total MNP concentrations of 0.5 g L^−1^. The experiments were conducted in 50 mM Tris buffer at pH 7.5. (d) Adsorption isotherm of PET microparticles to MNPs in 50 mM Tris buffer at pH 7.5. Error bars derive from three incubation experiments and supernatant analysis in triplicates (±SD). Recyclability of NBCs (e) and influence of the duration of storage on NBCs (f). Relative specific activity of the NBCs is compared to an equivalent free enzyme solution (both 0.072 g L^−1^ PETase) over different reuse cycles. The influence of a time dependent storage at 4 °C on the enzymatic performance of NBCs is compared to an equal free enzyme solution. Both experiments shown in (e) and (f) were conducted in 50 mM Tris buffer at pH 7.5 and incubated with 1 mM pNP-acetate in triplicates and each experiment was analyzed three times. The error bars are derived from the Gaussian error distribution.

Magnetic iron oxide nanoparticles used for these experiments are based on a synthesis route derived from Massart^40^ and have been characterized by Thomas *et al.*^41^ In short, the nanoparticles have a primary particle diameter of 10 nm. The nanoparticles are superparamagnetic with a high saturation magnetization of 84 A m kg^−1^, consist mainly of magnetite, and are uncharged at neutral pH.^41^ All experiments conducted in this study are described in the experimental section in the ESI.† The PETase purified with immobilized metal affinity chromatography (IMAC) to a purity of 80% (Fig. S1†) is used for the immobilization and mixed with the MNPs in different concentration ratios (Fig. 1b). The adsorption isotherm yields a *K*_D_ value of 0.075 g L^−1^ as a measure for the affinity of the His-tagged proteins to the nanoparticles (Fig. 1a and b). This dissociation constant indicates a high affinity and is in a similar range as binding affinities observed for His-tagged proteins on MNPs.^39^ Histidine based tags bind through the imidazole group to the iron oxide surface which can be especially observed by changes in the ring vibrations at 1199 and 1140 cm^−1^ upon adsorption of His_6_ peptides (Fig. S2†).^42^ An adaptation of the histidine coordination can also be observed by the binding of a fusion protein containing a histidine based tag (Fig. S3†).^32,43^ The bands at 1199, 1140 and 1050 cm^−1^ corresponding to ring vibrations significantly decrease which indicates an interaction with the iron oxide surface.^44^ The affinity is only slightly lower than the affinity of the novel immobilization tag presented by Zanker *et al.* for a comparable enzyme system.^32^ However, the affinity is significantly higher than the affinity of nonspecifically adsorbed enzymes to iron oxide nanoparticles.^37,38^ The maximum binding capacity is at 0.47 g g^−1^ which can be extrapolated from the Langmuir fit in Fig. 1b. The binding capacity of this enzyme is even slightly higher than the one observed by Zanker *et al.*^32^ but is similarly restricted to the amount of binding sites on the particle surface, which is dependent on the particle aggregation.^45,46^ Thus, a commercially available His-tag allows for the immobilization of enzymes on bare iron oxide nanoparticles with a high specific binding affinity. The particles aggregate at neutral pH, which was observed optically during the binding and separation process, makes a magnetic separation possible.^45^

This behavior is usually enhanced by the presence of proteins in solution.^45,47,48^ Here, a distinct increase in the particle number distribution to higher hydrodynamic diameters could be observed with increasing amounts of bound enzyme (Fig. 1c). Bare nanoparticles demonstrate a bimodal distribution with a high amount of dispersed particles around 20 nm which corresponds to the primary particle size of 10 nm (Fig. 1c).^41^ However, aggregates between 250 and 1000 nm were already visible for bare particles in Tris buffer at pH 7.5.

With increasing protein load, another fraction appeared at around 60–70 nm while the smaller fraction at 20 nm decreased. This behavior was already observed by Roth *et al.* and can be attributed to the protein corona forming around the bare nanoparticles.^38,49^ However, the increasing particle size distribution can also be an aggregation effect. With further increasing protein load, the smallest fraction disappeared while a fraction between 50 and 200 nm could be observed and the intensity of larger colloids between 200 and 1000 nm is increasing. This behavior has been observed for proteins bound to iron oxide nanoparticles with other affinity tags as well.^45^ The stability of small colloid fractions is destabilized by the presence of proteins and therefore the nanoparticles tend to aggregate and include the enzymes. However, this dynamic aggregation is usually reversible and does not significantly affect the enzymatic activity.^38^ Furthermore, the colloidal instability improves the recovery and therefore minimizes the loss of magnetic nanoparticles during a separation process with an applied magnetic field.^37^ The challenge is to combine these beneficial properties of a colloidal unstable system with the accessibility and the mass transfer properties of colloidal stable nanoparticles.

To demonstrate the advantages of the PETase immobilization on MNPs, we tested the reuse of the immobilized enzymes for different storage times up to two weeks at 4 °C and for the recycling with magnetic separation. Recycling the immobilized enzyme by magnetic separation is an up-and-coming concept. After ten cycles, still a high enzymatic activity of immobilized PETase was detectable (Fig. 1e). The high fluctuations can be explained as consequence of aggregation and deagglomeration effects with and without the magnetic field applied. After the particles settled down, the formation of agglomerates started again hindering enzymatic performance. After the fourth cycle, the relative specific activity of the NBCs fluctuates between 45–60% for the non-washed NBCs. Similar values can be observed for uncoated MNPs as carrier material for cellulose^37,50^ or ene-reductase.^32^

To analyze the influence of a static storage (4 °C) on the activity of the immobilized enzyme, we determined the relative specific activity after storing the NBCs for different time spans up to two weeks at 4 °C (Fig. 1f). The relative specific activity ranges between 40% and 70% but no trend to lower activities with storage time can be observed. This decrease in the specific activity may be caused by a possible instability of the PETase.^51^ Electrostatic interactions between the enzymes themselves can lead to a conformity change impeding the enzymatic activity. No degeneration or adsorption of 4-nitrophenol (pNP) was monitored for the blank MNP samples. We want to emphasize that 4-nitrophenol is only a common model system for PETase which has different requirements for the accessibility and mass transport than a larger microplastic particle.^24,52^ Not only the enzymatic degradation, but also the magnetic separation of microplastics is possible.^53,54^ Up to 70 g PET could be bound to 1 g MNP while a separation in a magnetic field was still possible (Fig. 1d). The magnetophoretic force on the MNP-PET complexes should be quite low in magnetic fields of around 100 mT which corresponds to the used NdFeB magnets. However, the magnetophoretic force is still sufficient to separate the complexes magnetically.

**Fig. 2:**
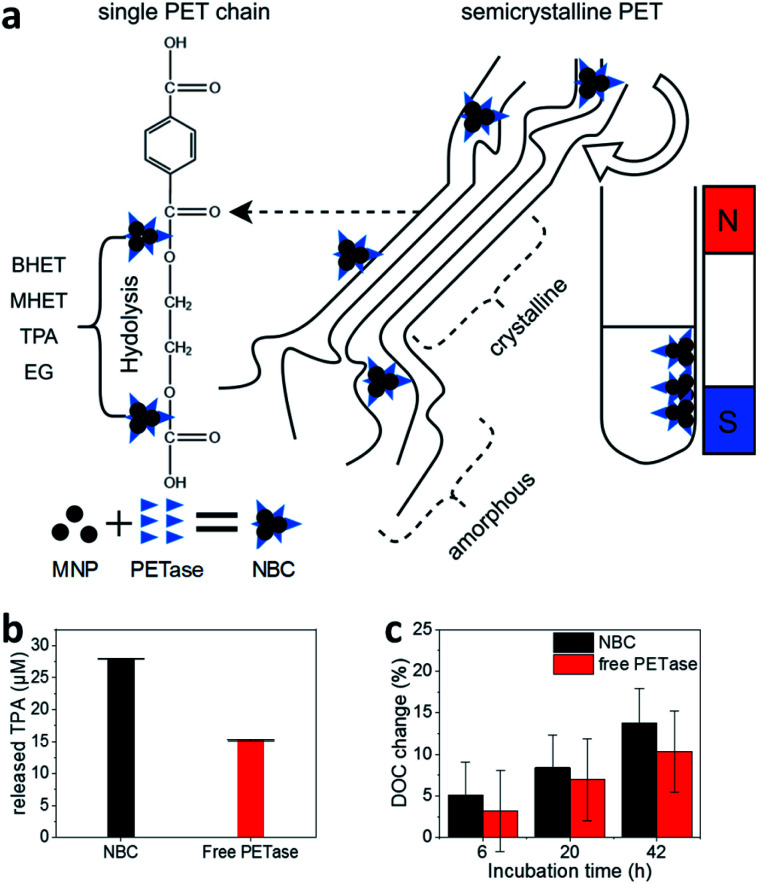
(a) Schematic illustration of the PET degradation in different crystalline regimes. Byproducts bis(2-hydroxyethyl) terephthalate (BHET) and mono-(2-hydroxyethyl)terephthalic acid (MHET) are abbreviated in the illustration. (b) Photometric estimation of the amount of released TPA. The supernatant of enzymatic degradation experiments was filtered (pore size 0.22 μM) and analyzed photometrically at 240 nm. The error bars were derived from three incubation experiments and supernatant analysis in triplicates (±SD). (c) Degree of crystallinity (DOC) change after enzymatic treatment. 20 ± 1 mg PET was incubated with 1.17 μM PETase in a free aqueous solution (red bar) and NBCs with an equivalent enzyme concentration (black bar). All experiments were conducted at room temperature. DOC was derived from DSC measurements (Fig. S4†). Each experiment was performed in triplicates and measured once (±SD).

While pNP assays to measure the enzymatic activity of enzymes have advantageous characteristics such as the simple handling and easy adaptability, enzymes and NBCs need to be evaluated towards their ability to degrade MPs. We investigated the release of PET degradation products with UV/Vis spectroscopy in order to estimate the enzymatic activity towards PET-based substrates. NBCs showed an enzyme activity of around 0.7 μmol g^−1^ h^−1^ (Fig. 2b) which is in good agreement with the measured activity in literature.^21^ The release of TPA and therefore the enzymatic activity of NBCs was even higher than the one obtained with the free enzyme. This degradation of a PET substrate makes a strong case for the usage of NBCs to degrade MP.

With NBCs, not only the enzymatic activity but also the mass transport is important. How can an NBC enzymatically attack a semi-crystalline material? In this context, we studied the properties of MPs before and after partial enzymatic degradation. The crystallinity of PET can be analyzed with Raman spectroscopy. Peaks at 1119 cm^−1^ and 1096 cm^−1^ refer to the C–O–C stretching modes. We compared several PET sources towards their crystallinity with Raman spectroscopy and DSC (Fig. S4–S7†). Most PET sources showed a crystallinity between 5–30% with the granulate and powder showing the lowest crystallinity Fig. S4.† ^55,56^ The same trend is visible from the comparison of the C–O–C stretching modes with Raman spectroscopy (Fig. S7†). The hard plastic PET bottles show the highest degree of crystallinity (DOC) followed by the soft bottles and the PET film. The same trend can be observed from DSC results as well as from Raman spectroscopy analysis of C–O–C vibrations. The change of crystallinity was monitored over three different incubation times (Fig. 2c). A slight increase in the crystallinity of the PET samples by the addition of buffer only or MNPs can be derived from DSC. A significant increase of the DOC can be observed after incubation of PET with NBCs and free enzymes. Longer incubation times led to higher crystallinities, which means less crystalline PET can be degraded faster than crystalline compartments. Furthermore, the presence of NBCs led to higher crystallinities than free enzymes. This behavior might be due to the more limited access of the NBCs to the PET substrate compared to the free enzyme. The results are in good agreement with other PET degradation experiments by free PETase^52^ and this is also known to occur with other substrate enzyme systems such as wheat straw and cellulose where steric hindrance challenges enzymatic activity (Fig. 2a).^57^ Nevertheless, magnetic nanoparticles can be used as carriers for PETase which allow for a fast degradation of PET and the recycling of enzymes.

## Conclusions

We revealed a promising site-directed immobilization strategy for PETase on bare magnetic nanoparticles *via* a His-tag. The immobilization of PETase (PETaseS238F/W159H)^52^ on iron oxide nanoparticles can be realized with high enzyme loads of up to 0.47 g g^−1^ and high affinity. These nanobiocatalysts (NBC) can be recycled magnetically and maintain around 50% of their initial enzymatic activity after 10 cycles. The NBCs can be magnetically separated with relatively small losses and are able to degrade PET substrates. We observed a crystallinity increase of 15% after 42 hours of incubation with these NBCs at room temperature. Here, the NBCs led to higher crystalline microparticles than the free enzymes which can be accounted to the limited access and the higher activity. Furthermore, it could be shown that the NBC outperformed a comparable free PETase solution after 42 h in the context of PET microparticle decomposition. Besides, magnetic nanoparticles have a high magnetic fishing potential of the MNPs towards PET and are able to carry ∼70 times their own mass of GF-PET. This work paves the way for new strategies for microplastic reduction based on MP-degrading enzymes immobilized on magnetic nanocarriers.

## Author contributions

Sebastian P. Schwaminger: conceptualization, writing – original draft, validation, visualization, writing – review & editing. Stefan Fehn: data curation, formal analysis, investigation, methodology, visualization, writing – original draft. Stefan Rauwolf: investigation, methodology, writing – review & editing. Tobias Steegmüller: data curation, investigation, methodology, visualization, writing – review & editing. Hannes Löwe: investigation, methodology, writing – review & editing. Katharina Pflüger-Grau: conceptualization, writing – review & editing. Sonja Berensmeier: project administration, resources, supervision, writing – review & editing.

## Conflicts of interest

There are no conflicts to declare.

## Supplementary Material

NA-003-D1NA00243K-s001
