# Supplementary material for: Immobilization of PETase enzymes on magnetic iron oxide nanoparticles for the decomposition of microplastic PET
Source: Nanoscale Adv. 2021 Jun 14;3(15):4395–9. doi: 10.1039/d1na00243k (PMC9417550; doi:10.1039/d1na00243k)
Supplement: NA-003-D1NA00243K-s001 [file NA-003-D1NA00243K-s001.pdf]

Electronic Supplementary Material (ESI) for Nanoscale Advances.
This journal is © The Royal Society of Chemistry 2021

Electronic Supplementary Material

Immobilization of PETase enzymes on magnetic iron oxide nanoparticles for the decomposition of microplastic PET

Sebastian P. Schwaminger,[a,*] Stefan Fehn,[a] Tobias Steegmüller,[a] Stefan Rauwolf,[a] Hannes Löwe,[b] Katharina Pflüger-Grau,[b] Sonja Berensmeier[a,*]

[a]Bioseparation Engineering Group, Department of Mechanical Engineering, Technical University of Munich, Garching, Germany
[b]Systems Biotechnology, Department of Mechanical Engineering, Technical University of Munich, Garching, Germany

Experimental

All reagents used are commercially available, have a purity of >95% (high-performance liquid chromatography, HPLC grade), and were used as received without further purification. PETase was expressed essentially as described by Austin et al.[1] The gene coding PETase corresponds to the gene of *Ideonella sakaiensis* strain 201-F6 (Genbank GAP38373.1). The vector plasmid pET-21b(+)-Is-PETase-W159H-S238F (Addgene ID: 112203) was extracted and transformed into *E. coli* BL21 (DE3) by the method of Chung et al.[2] For PETase expression, 5 ml of 2xYT-medium (16 g L^{-1} trypton/pepton, 10 g L^{-1} yeast extract, 5 g L^{-1} NaCl) with 150 mg L^{-1} ampicillin was inoculated with *E. coli* BL21 carrying pET-21b(+)-Is-PETase-W159H-S238F and incubated over night at 37 °C. The culture was transferred to shake flasks the following day and incubated at 37 °C for approximate 3 h at 200 rpm until an optical density (OD_{600}) between 0.6-0.8 was reached. Next, the cultures were induced with 0.1 mM IPTG and incubated at 16 °C for 20 h at 200 rpm. Cells were harvested by centrifuging at 3200xg for 20 min and subsequently the supernatant was discarded. The remaining pellets were stored at -20 °C. For cell disruption the previously obtained pellets were dissolved in 5 mL equilibration buffer (20 mM Na_3PO_4, 500 mM NaCl) by vortexing. Protease inhibitor was added to each suspension. The tubes with the dissolved cells were put on ice and disrupted mechanically by a French press (Julabo GmbH, Seelbach) at 1.8 kbar and 8 °C. The lysate was centrifuged at 17000xg to get rid of cell debris. From the obtained lysate PETase was purified by an Immobilized Metal Ion Chromatography (IMAC) system (Äkta Explorer, GE Healthcare, Chicago). The analytical detection of PETase was carried out by sodium dodecyl sulfate polyacrylamide gel electrophoresis (SDS-PAGE) with a 5 % collection and a 12 % separation gel (160 V, 300 mA and 30 W for 60 min). *The E. coli* lysate and the purified PETase enzyme after IMAC elution were compared on the gel. The success of the PETase purification was determined by comparing the contrast intensity between the band of interest (~30 kDa) and other bands on the gel. Therefore, the Coomassie-stained SDS-PAGE (0.1% Coomassie, 10% acetic acid, 40% ethanol) was analysed in a biomolecular imager (Amersham Typhoon, GE Healthcare, Chicago).

Before quantifying the corresponding PETase concentrations, the purified lysate was rebuffered in Tris(hydroxymethyl)aminomethane(Tris)-HCl buffer at pH 7.5. The lysate was filled in tubes with a pore size of 10 kDa and centrifuged at 4 °C and 3200 g for 20 min. The filtered liquid was disposed while the remaining liquid containing the target protein was refilled again with buffer. This procedure was repeated three times. Afterwards, each remaining liquid containing PETase was aliquoted in 200 µL portions and dried at ~80 °C.

Colorimetric protein quantification was determined by a bicinchoninic acid (BCA) assay (Merck KGaA, Darmstadt, Germany) according to the manufacturer's user guide. Analysis was performed at 562 nm with an Infinite M200 Microplate reader (Tecan Deutschland, Germany). The calibration curve was calculated by averaging the corresponding triplicates and subtracting the buffer blank. Protein concentrations were calculated from the corresponding linear relationship of absorption and concentration of the standard. The experiments were performed in triplicates. Green fluorescent protein (GFP) without (woT) and with an (RH)4-tag (containing four histidines and four arginines) has been synthesized and purified as described by Zanker et al.[3] Histidine hexapeptides have been obtained from ? and mixed with magnetic nanoparticles in a range of 1:1 (w/w) with 0.5 g L^{-1} nanoparticles in a 1 mL suspension for 30 min at 25 °C under vigorous shaking.

Magnetite was synthesized following the co-precipitation of Fe^{2+} and Fe^{3+} in alkaline environment as described by Roth et. al.[4–6] Briefly, 35.0 g of iron (II) chloride and 86.4 g of iron (III) chloride, obtained from Sigma-Aldrich, were dissolved, and mixed in 200 mL of ddH_2O. 1 M NaOH (Fluka) was prepared with ddH_2O and filled in a stirred tank glass reactor under a nitrogen atmosphere. Subsequently, the salt solution was added dropwise, which

immediately formed a black precipitate. After complete addition, the reaction was continued for half an hour under constant conditions. The resulting particles were washed several times with ddH_2O until a conductivity of less than 200 µS cm^{-1} was reached.

Adsorption isotherms of PET on magnetite were determined by dry weight. Different PET concentrations (0-100 g L^{-1}) were incubated with 1 g L^{-1} iron oxide solution. The particles were separated magnetically. Therefore, a NdFeB magnet Q-40-40-20-N from Webcraft GmbH (N42) was placed next to the reaction vessel. The same magnet and separation procedure was used for all magnetic separations. The supernatant was removed with a pipette, dried in an oven and weighted.

PETase was immobilized on magnetite particles by mixing 1 g L^{-1} magnetite solution (1 mL) with 1 mL PETase in different concentrations between 0 - 1 g L^{-1}. Incubation was performed at 30 °C and 700 rpm for 1 h. A magnetic holder was used to separate the liquid phase from the solid phase. The protein concentration in the liquid phase (equilibrium concentration c*) was analyzed as previously described by a BCA-Assay and photometrically at 230 nm.

Moreover, the adsorption behavior was quantified by Dynamic Light Scattering (DLS) analysis. 100 µL of the NBCs with different loads were diluted at the rate of 1:10 in buffer until a final NBC concentration with 0.05 g L^{-1} with the corresponding loads was reached. Afterwards, they were analyzed with a Particle Analyzer (Beckmann Coulter, Krefeld). The measurements were conducted in triplicates consisting of two cycles of ten individual measurements. For size distribution analysis both measurements were averaged per experiment.

Enzymatic activity measurements were performed by incubation of different enzyme concentrations with 1 mM 4-nitrophenyl-acetate (pNP-acetate) in 50 mM Tris buffer at pH 7.5. Before incubation with the enzyme, the pNP-acetate solution was solved at 37 °C for 30 min. The 4-nitrophenol concentration was monitored at 410 nm with UV/Vis spectroscopy. Direct comparison of enzyme kinetics with 1 µM of immobilized PETase and PETases in free enzyme solution was performed with 1 mM pNP acetate and monitored for several hours. For the recycling experiment, washing (with magnetic holder) and enzyme activity steps were alternated.

For PET characterization five different PET bulk materials were investigated. Glass fiber reinforced (GF-)PET granulates (30 % GF (w/w), Sigma-Aldrich, Saint Louis, USA) were pulverized to PET powder by a cryogenic mill (Pulverisette 14, Fritsch, Germany) at ~12000 rpm. The milling process was repeated several times. A sieve ring with a grain diameter of 0.5 mm was used during pulverization of the PET granulate. In addition, 5 µm thick PET film (Sigma-Aldrich, Saint Louis, USA) and the bottle wall of a commercially available soft and hard drinking bottles were analyzed.

For enzymatic treatment, 250 µL PETase solution (0.1 g L^{-1}) was incubated with 250 µL MNPs (1 g L^{-1}) for 1 h at 30 °C. Afterwards, the supernatant was randomly analyzed to calculate the local load of this immobilization experiment. The NBCs were washed twice and stored at pH 7.5 (500 µL of 50 mM Tris buffer). The NBCs, free enzyme solutions containing an equal PETase concentration to the load on the NBCs, 500 µl bare MNP solutions (0.5 g L^{-1}) and 500 µL buffer were incubated each with 20 ± 1 mg GF-PET powder. The incubation was carried out under continuous stirring at 700 rpm for 6 h, 20 h, or 42 h, respectively. After incubation, the samples were centrifuged at 17000 rpm for 30 min and supernatant was filtered (pore size 0.22 µm) and analyzed with UV/vis spectroscopy. The residual PET samples were dried at room temperature. Enzymatic treatments were carried out in triplicate being analyzed three times each. The error bars represent the respective standard deviations.

The crystallinity of the (GF)-PET samples was determined by differential scanning calorimetry (DSC 204, Netzsch, Waldkraiburg). MNP- or NBC-treated (GF-)PET samples were separated with a magnetic holder and the

supernatant was analyzed. For DSC measurements, the PET samples were heated from room temperature to 300 °C at a rate of 10 K min^{-1} and held for 60 s at this temperature. The melting heat enthalpy ΔH was obtained by the integral of the melting peak around 250 °C. The degree of crystallinity (%DOC) is calculated with the following equation[7]:

$$\%DOC = \left[\frac{\Delta H_m - \Delta H_c}{140\ [\frac{J}{g}] * (1 - 0.3)}\right] \times 100 = \left[\frac{\Delta H_m - \Delta H_c}{98\ [\frac{J}{g}]}\right]$$

Raman spectra for further crystallinity analysis were recorded by a Raman spectrometer (SENTERRA, Bruker Optics GmbH, Ettlingen). Therefore, samples containing MNPs or NBCs were separated by a magnetic holder. The spectra were measured with a laser at a wavelength of 488 nm and laser power of 4 mW with a 50x objective. The integration time for each measurement was 60 s. Each spectrum was measured twice and averaged. The crystallinity of the polymer material was determined by deconvolution of the Raman spectra between 1085 cm^{-1} and 1125 cm^{-1}. The curves were deconvoluted by a Voigt fit into and 3 - 5 single integrals around 1090 cm^{-1}, 1096 cm^{-1}, 1103 cm^{-1}, 1113 cm^{-1} and 1119 cm^{-1}. To determine and compare the influence on the crystallinity between pure PET and GF-PET samples, the relative peak area p_{rel} was calculated by putting the peaks in relation [8]:

$$p_{rel} = \frac{p_{\sim 1090} + p_{\sim 1096}}{p_{\sim 1090} + p_{\sim 1096} + p_{\sim 1113} + p_{\sim 1119}}$$

Furthermore, the Raman spectra around 1725 cm^{-1} of the different PET samples were analyzed in relation to their full width at half maximum (FWHM). Likewise, the peaks around 1725 cm^{-1} and 1714 cm^{-1} were deconvoluted by a Voigt fit. For both analysis methods two different spots from each sample were measured and averaged. The error bars represent the standard deviation from the fitting parameters of each experiment.

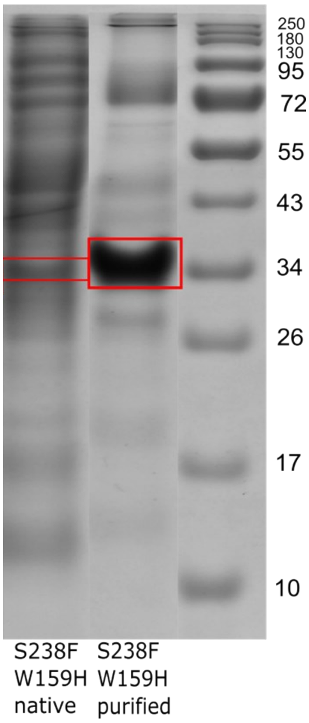

Fig S1 SDS-PAGEs of the native lysate and the purified PETase after IMAC. The red borders mark the proteins at a size of approximately 30 kDa corresponding to the molecular size of $PETase^{S238F/W159H}$.

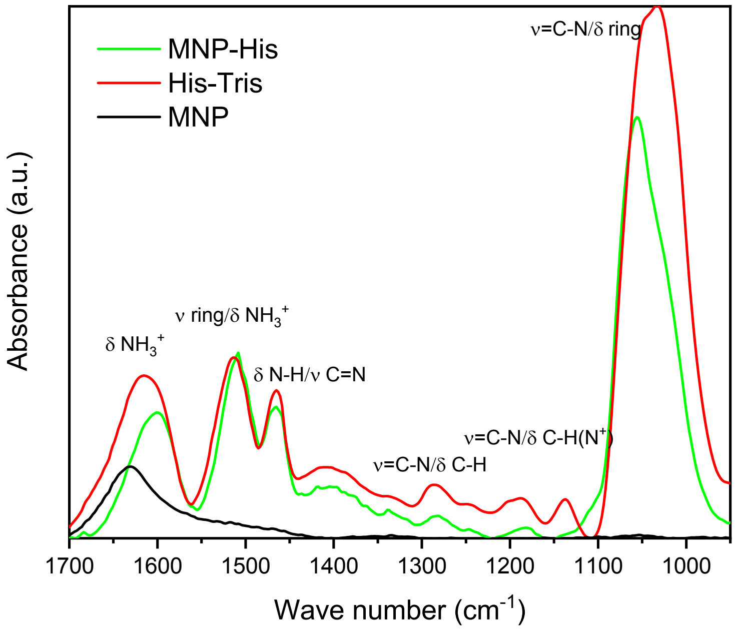

Fig S2 Infrared spectra of a histidine hexapeptide and bare magnetic nanoparticles as well as nanoparticles with bound histidine precipitated on an ATR crystal.

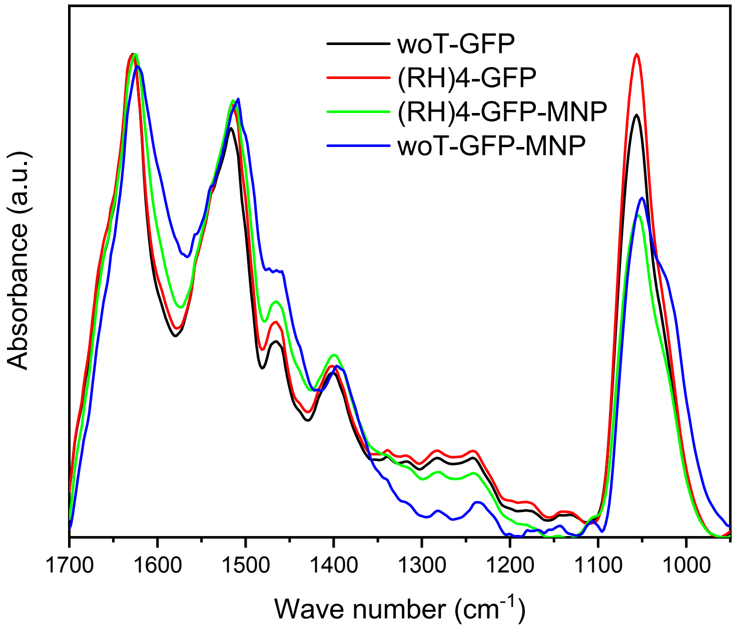

Fig S3 Infrared spectra of tagged and untagged GFP as well as nanoparticles with bound protein precipitated on an ATR crystal.

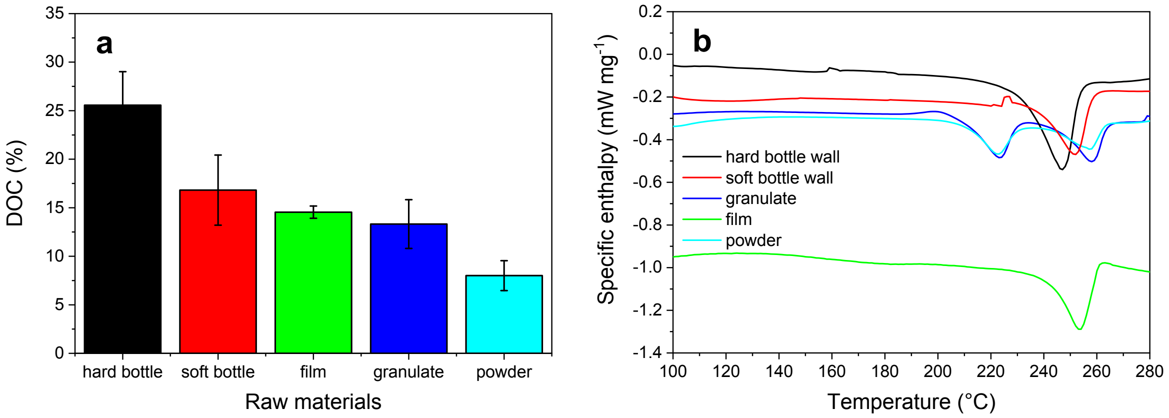

Fig S4 (a) Degree of crystallinity (DOC) of different bulk materials derived from DSC experiments (b). Each material was heated from room temperature to 300 °C at a rate of 10 K min^{-1}. The samples were taken from the respective basic population and measured in triplicates (±SD).

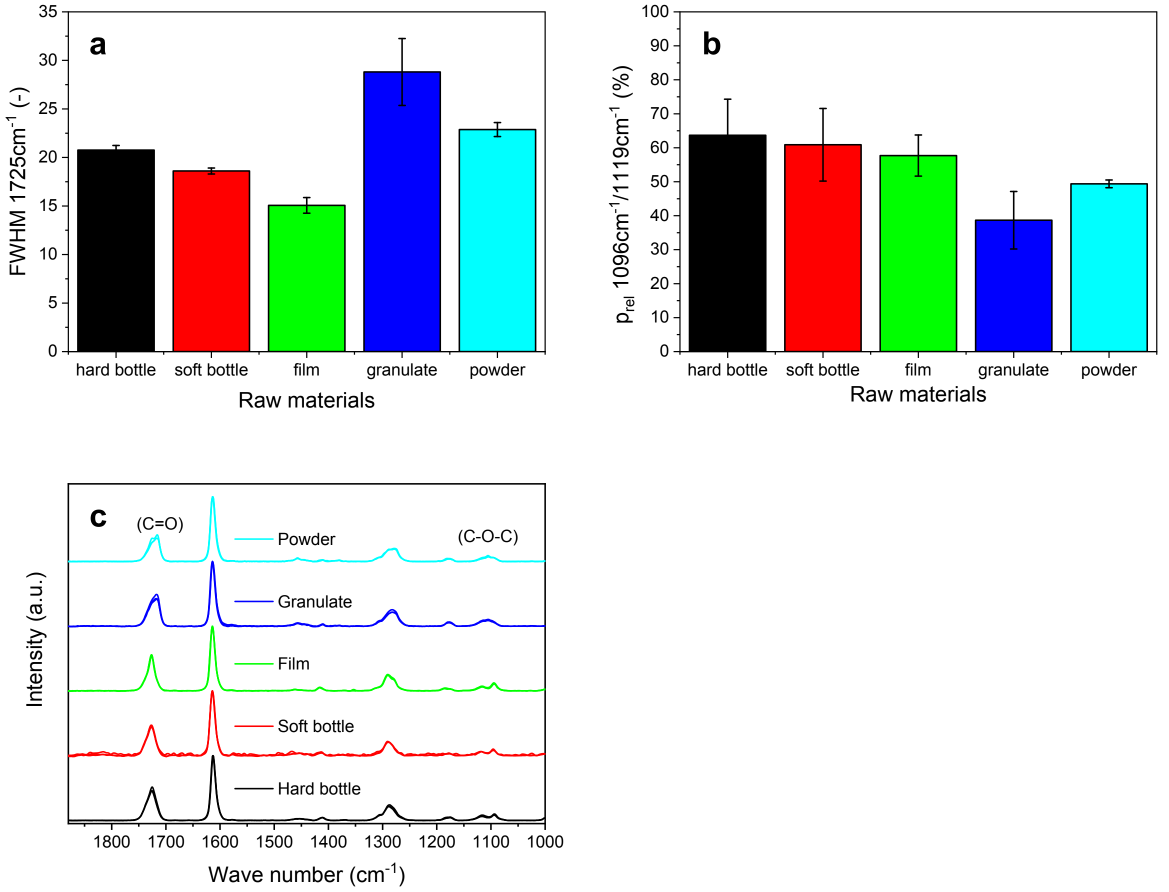

Fig S5 Full width at half maximum (FWHM) (a) and relative peak area (b) of different raw materials by analysis of Raman signals around 1725 cm^{-1} and 1096 cm^{-1}/1119 cm^{-1}. A lower FWHM and a higher relative peak are related to a higher state of crystallinity for the particular material. The averaged spectra of three different duplicate Raman spectra were used for the fitting procedure. The standard deviation is represented for three corresponding experiments (±SD). (c) Full Raman spectra of different PET materials (three experiments each) irradiated with a 488 nm laser.

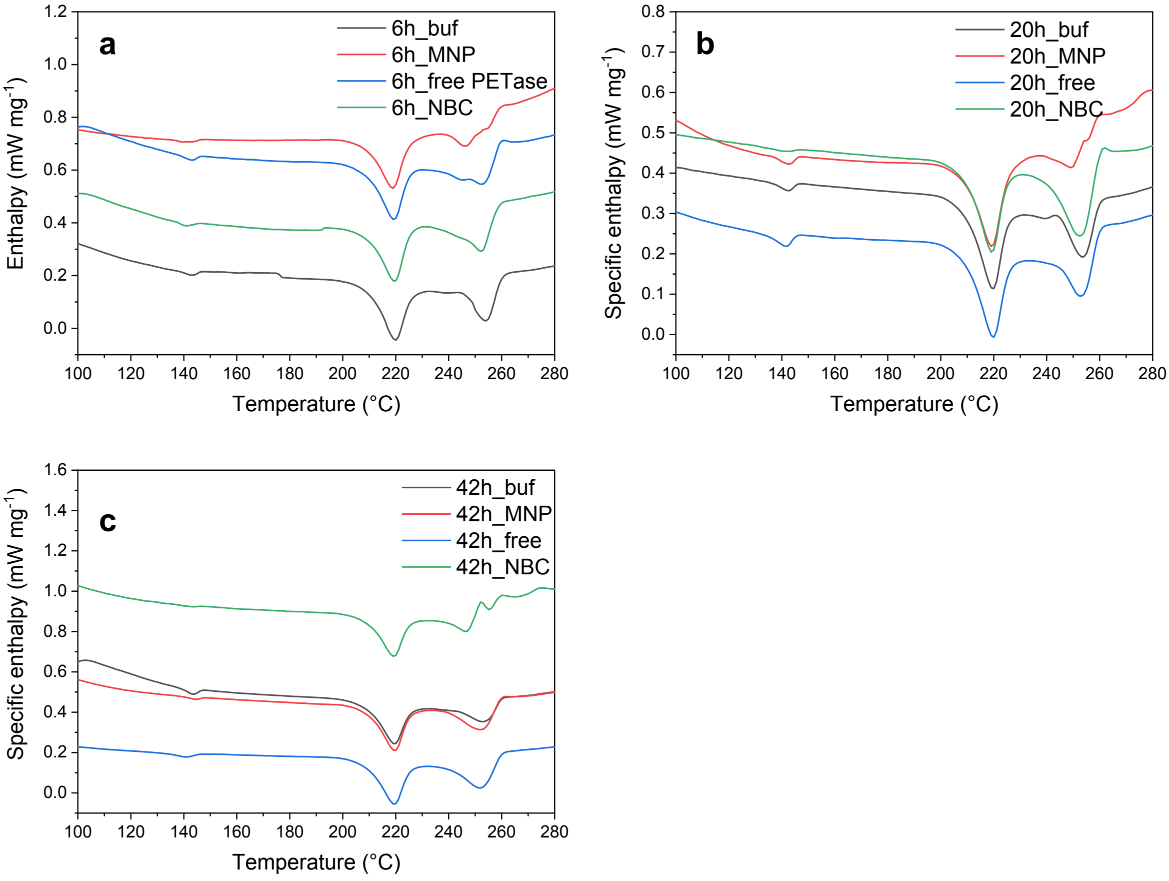

Fig S6 DSC of PET after 6 h (a), 20 h (b) and 42 h (c) from 100 -280 °C with a heating rate of 10 K min^{-1}. PET was incubated with buffer (black), with MNP (red), with free PETase (blue) and with NBCs (green).

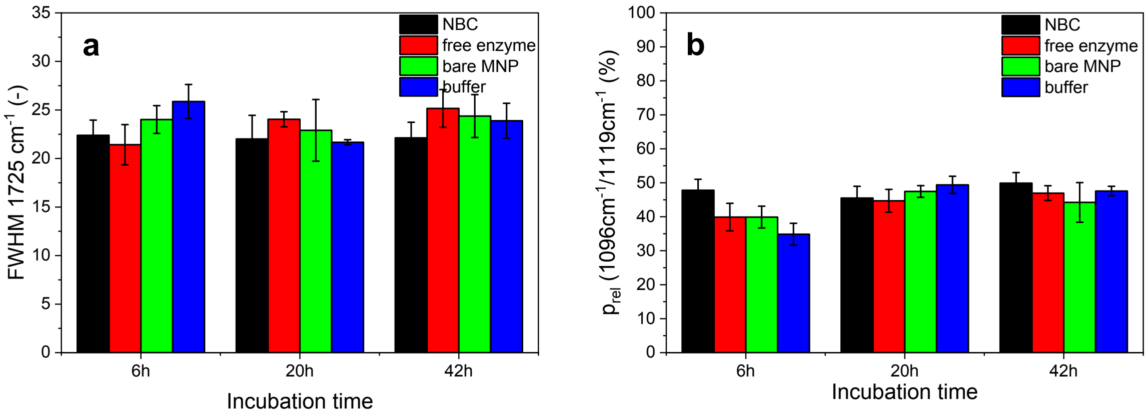

Fig S7 FWHM (a) and relative peak area (b) after enzymatic treatment. The GF-PET powder was treated with 1.17 µM PETase$^{S238/W159H}$ (blue bars) and NBCs with an equivalent enzyme concentration (gray bars) for 42 h. All experiments were conducted at room temperature. The standard deviation is represented for three independent experiments (±SD).

References

1 H. P. Austin, M. D. Allen, B. S. Donohoe, N. A. Rorrer, F. L. Kearns, R. L. Silveira, B. C. Pollard, G. Dominick, R. Duman, K. El Omari, V. Mykhaylyk, A. Wagner, W. E. Michener, A. Amore, M. S. Skaf, M. F. Crowley, A. W. Thorne, C. W. Johnson, H. L. Woodcock, J. E. McGeehan and G. T. Beckham, *Proc. Natl. Acad. Sci. U. S. A.*, 2018, **115**, E4350-E4357.

2 C. T. Chung, S. L. Niemela and R. H. Miller, *Proc. Natl. Acad. Sci. U. S. A.*, 1989, **86**, 2172–2175.

3 A. A. Zanker, N. Ahmad, T. H. Son, S. P. Schwaminger and S. Berensmeier, *Biotechnol. J.*, 2020, e2000366.

4 H.-C. Roth, S. P. Schwaminger, M. Schindler, F. E. Wagner and S. Berensmeier, *J. Magn. Magn. Mater.*, 2015, **377**, 81–89.

5 J. A. Thomas, F. Schnell, Y. Kaveh-Baghbaderani, S. Berensmeier and S. P. Schwaminger, *Chemosensors*, 2020, **8**, 17.

6 R. Massart, *IEEE Trans. Magn.*, 1981, **17**, 1247–1248.

7 Y. Kong and J. N. Hay, *Polymer*, 2002, **43**, 3873–3878.

8 A. J. Melveger, *J. Polym. Sci. A-2 Polym. Phys.*, 1972, **10**, 317–322.
